# Severe Cutaneous Adverse Reaction to Intraperitoneal Vancomycin: A Case of DRESS (Drug Reaction with Eosinophilia and Systemic Symptoms) Syndrome

**DOI:** 10.7759/cureus.90704

**Published:** 2025-08-21

**Authors:** Kelly Andrea Arenas Sanchez, Alejandra Maria Paguaga Morales, Luis Fernando Muñoz Chávez, Leticia Lopez Carreola, Sandra I Dominguez Valdez

**Affiliations:** 1 Internal Medicine, Centro Medico Nacional 20 de Noviembre, Mexico City, MEX

**Keywords:** drug rash, drug reaction with eosinophilia and systemic symptoms (dress) syndrome, skin lesions, vancomycin-induced dress syndrome, vancomycin reaction

## Abstract

Drug Reaction with Eosinophilia and Systemic Symptoms (DRESS) syndrome is a rare but potentially life-threatening hypersensitivity reaction to certain medications, including allopurinol (a xanthine oxidase inhibitor), antiepileptics (e.g., phenytoin, carbamazepine), and antibiotics (e.g., vancomycin). It typically presents within 2-12 weeks after administration of the offending drug. DRESS causes a characteristic rash, fever, renal alterations (acute kidney injury with associated findings of hematuria and proteinuria), hematologic alterations (eosinophilia, leukocytosis with the presence of atypical lymphocytes, and occasionally anemia or thrombocytopenia), and hepatic involvement, which represents the main organ manifestation, characterized by hepatitis with elevated transaminases. It is a potentially life-threatening multiorgan disorder. Although vancomycin is a known causative agent, DRESS associated with intraperitoneal vancomycin remains extremely rare. We report the case of a 59-year-old male with a history of type 2 diabetes, benign prostatic hyperplasia, systemic arterial hypertension, and KDIGO (Kidney Disease: Improving Global Outcomes) stage 5 chronic kidney disease, on peritoneal dialysis via a Tenckhoff catheter, who developed generalized dermatosis, systemic symptoms, and laboratory findings consistent with DRESS, 25 days after initiating intraperitoneal vancomycin for bacterial peritonitis. We performed a detailed clinical examination of the pruritic, violaceous maculopapular rash presented by the patient, along with laboratory analysis, which showed eosinophilia, elevated liver enzymes, and atypical lymphocytosis on peripheral blood smear. The diagnosis was confirmed using the Registry of Severe Cutaneous Adverse Reactions (RegiSCAR) scoring system and supported by histopathological findings from a skin biopsy, which revealed lymphocytic and eosinophilic inflammation. The patient responded favorably to withdrawal of the drug, as well as systemic corticosteroid therapy, with both clinical and laboratory improvement. This case highlights the need to consider intraperitoneal vancomycin as a potential trigger for DRESS, particularly in patients with impaired renal clearance. Prompt recognition, withdrawal of the offending agent, and initiation of immunosuppressive therapy are essential to reducing morbidity and preventing severe complications.

## Introduction

Drug Reaction with Eosinophilia and Systemic Symptoms (DRESS) syndrome is a severe drug-induced hypersensitivity reaction characterized by delayed onset, prolonged course, and multiorgan involvement, with an estimated mortality rate ranging from 10% to 20% [1,2]. Its pathophysiology is multifactorial and involves genetic predisposition (such as specific human leukocyte antigen (HLA) haplotypes), immune dysregulation, and viral reactivation, particularly of human herpesvirus 6 (HHV-6) [1,3].

Clinically, DRESS typically presents with fever, cutaneous rash, lymphadenopathy, eosinophilia, and abnormalities in the liver, hematologic system, or kidneys, among others. Diagnosis requires a high index of suspicion, mainly when symptoms occur between two and eight weeks after exposure (normal duration 2-12 weeks [4]) to the causative drug. Scoring systems such as the Registry of Severe Cutaneous Adverse Reactions (RegiSCAR) criteria have been developed to classify cases as definite, probable, or possible [2-4].

Multiple drug classes have been associated with DRESS, including anticonvulsants, allopurinol, sulfonamides, antituberculosis agents, antivirals, and antibiotics. Among the latter, vancomycin has emerged as a major culprit, with an increasing number of cases documented in the literature [5-8]. Recent studies suggest an average latency of 21 days and a higher risk of renal involvement in vancomycin-induced DRESS [6,7].

Despite extensive documentation of DRESS associated with intravenous vancomycin, reports regarding its intraperitoneal administration are scarce. This route, commonly used in patients undergoing peritoneal dialysis, may lead to sustained plasma concentrations due to slow but continuous absorption across the peritoneal membrane, potentially contributing to systemic toxicity [9].

We present the case of a male patient with end-stage renal disease on continuous peritoneal dialysis who developed DRESS syndrome following intraperitoneal vancomycin administration. This report aims to highlight this uncommon presentation and emphasize the importance of close clinical monitoring and timely diagnostic and therapeutic interventions in patients with predisposing risk factors.

## Case presentation

A 59-year-old male with a past medical history of type 2 diabetes, prostate enlargement, systemic arterial hypertension, and chronic kidney disease (KDIGO (Kidney Disease: Improving Global Outcomes) stage 5) on replacement therapy based on peritoneal dialysis using a Tenckhoff catheter. The patient developed bacterial peritonitis for which he was treated with intraperitoneal vancomycin 1 g every 48 hours for eight days.

**Figure 1:**
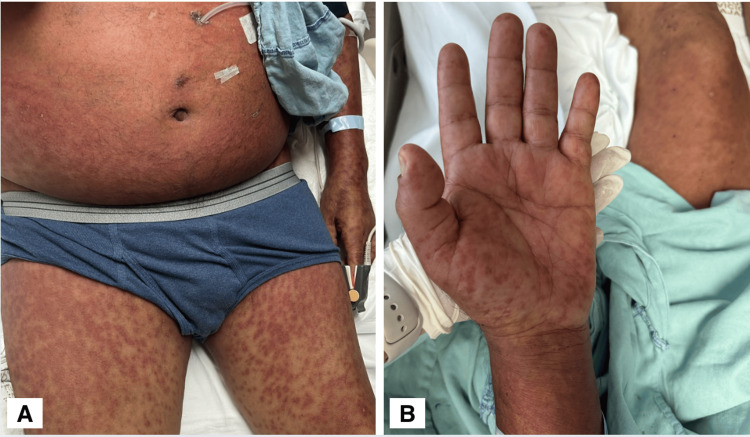
Dermatosis affecting the anterior trunk, upper and lower extremities, characterized by multiple plaques composed of violet-colored macules which converge to form larger plaques. A. Symmetric, coalescent, and confluent erythematous maculopapular rash involving the anterior trunk and lower extremities. B. Poorly demarcated, diffuse erythematous macules with a reticulated distribution on the palm and forearm of the left upper extremity, without desquamation.

Twenty-five days after the first application of vancomycin, the patient presented to the emergency room with a generalized dermatosis affecting the face, anterior and posterior trunk, upper and lower extremities, characterized by multiple plaques composed of violet-colored macules that disappeared with acupressure with fine adherent scales of 3-10 mm in diameter, which converged to form larger plaques, alternating with pustules, with pruritic symptoms (Figure 1), accompanied by headache, general malaise, and arterial hypotension. Organic liver involvement was reported with a remarkable eosinophilia, and he was hospitalized. Laboratory results are given in Table 1. Vancomycin levels were not measured due to a lack of availability.

**Table 1:** Laboratory test results

Test	Patient Values at Admission	Post-treatment Patient Values	Reference Ranges
Leukocytes	19.73	8.9	5000-10,000/mm^3^
Neutrophils	14.79	4.57	1400–6500/mm³
Lymphocytes	3.12	1.86	1000–3400/mm³
Eosinophils	2.99	0.2	0-700/mm^3^
Hemoglobin	10.7	12.1	12-16 g/dL
Hematocrit	31	38.3	37-47%
Mean corpuscular volume	96	83	82-96 fL
Mean corpuscular hemoglobin	33.1	27.9	27-31 pg
Platelets	136	140	150,000-450,000/mm^3^
Glucose	514	125	74-106 mg/dL
Creatinine	16.63	1.8	0.1-1.3 mg/dL
Urea	229	19.2	19-49 mg/dL
Sodium	132	136	132-146 mEq/L
Potassium	4.4	4.0	3.5-5.5 mEq/L
Chloride	92	108	99-109 mEq/L
Calcium	7.9	9.40	8.3-10.6 mg/dL
Phosphorus	6.2	3.30	2.4-5.1 mg/dL
Magnesium	2.11	2.02	1.3-2.7 mg/dL
Alanine transaminase	107	47	10-49 U/L
Aspartate aminotransferase	42	32	<34
Lactate dehydrogenase	684	147	120-146 UI/L
Alkaline phosphatase	222	129	45-120 U/L
International normalised ratio	1.14	1.10	0.8-1.1

**Figure 2:**
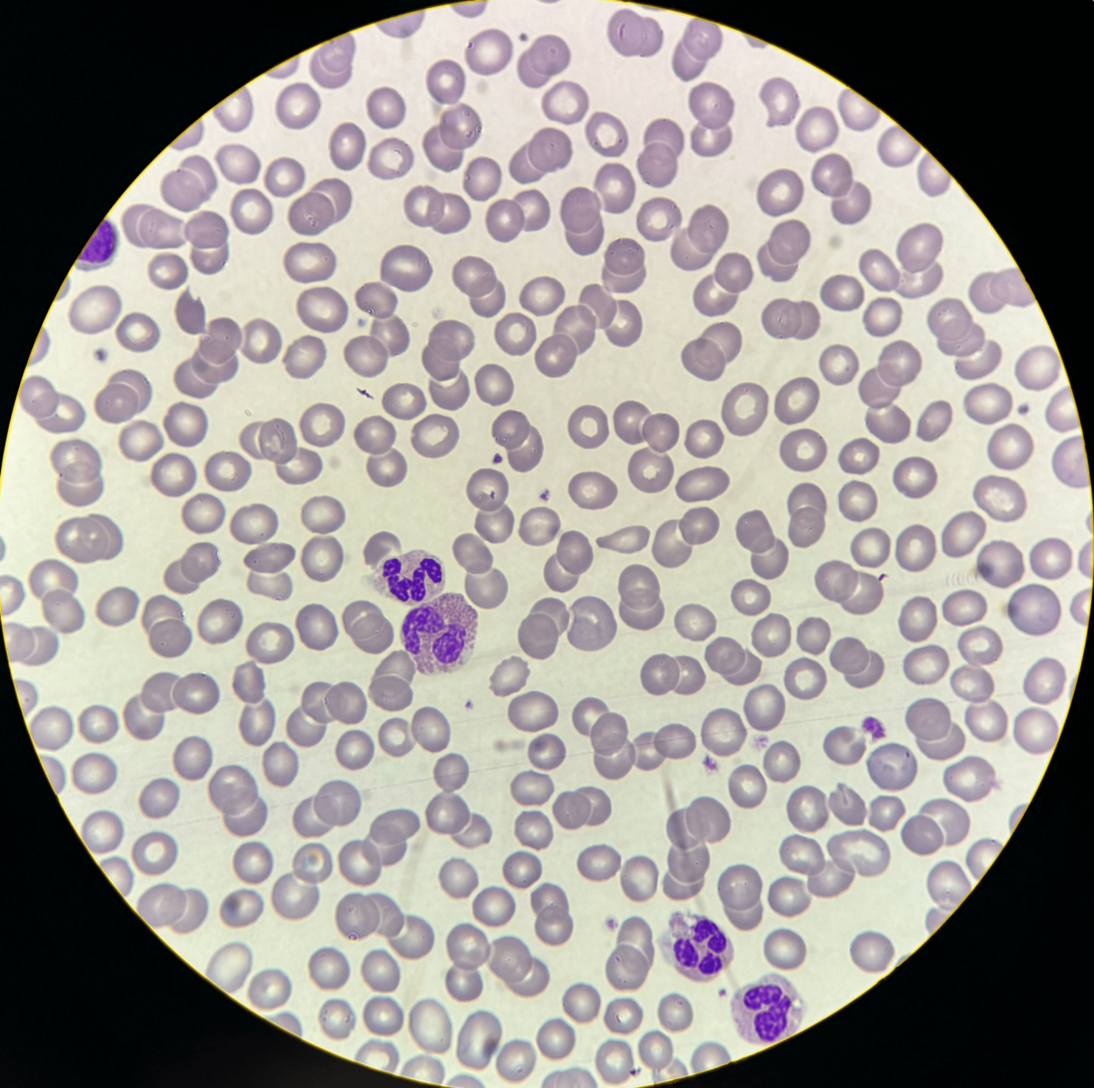
Peripheral blood smear showing atypical lymphocytes and eosinophils

**Figure 3:**
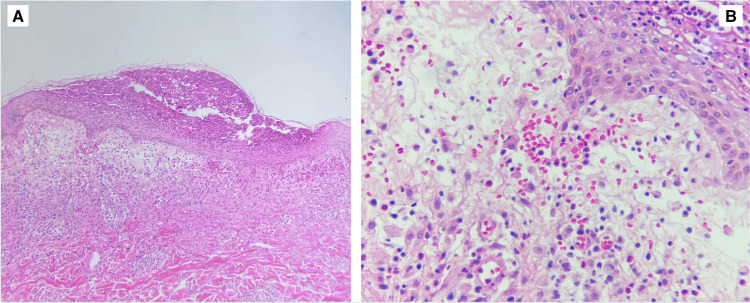
Skin biopsy A. Mild interpapillary edema associated with a mixed inflammatory infiltrate in the superficial dermis. B. Superficial dermis exhibits edema, vascular congestion, and a mixed inflammatory infiltrate with occasional eosinophils.

The patient's clinical presentation, drug history, eosinophilia, and the presence of atypical lymphocytes in a peripheral blood smear (Figure 2) resulted in a RegiSCAR score of 7, which was considered definitive for the diagnosis of DRESS. Additionally, a skin biopsy was performed using a 5mm punch of the dermatosis in the abdomen, which revealed superficial perivascular inflammatory infiltrates composed of lymphocytes and eosinophils (Figure 3).

**Figure 4:**
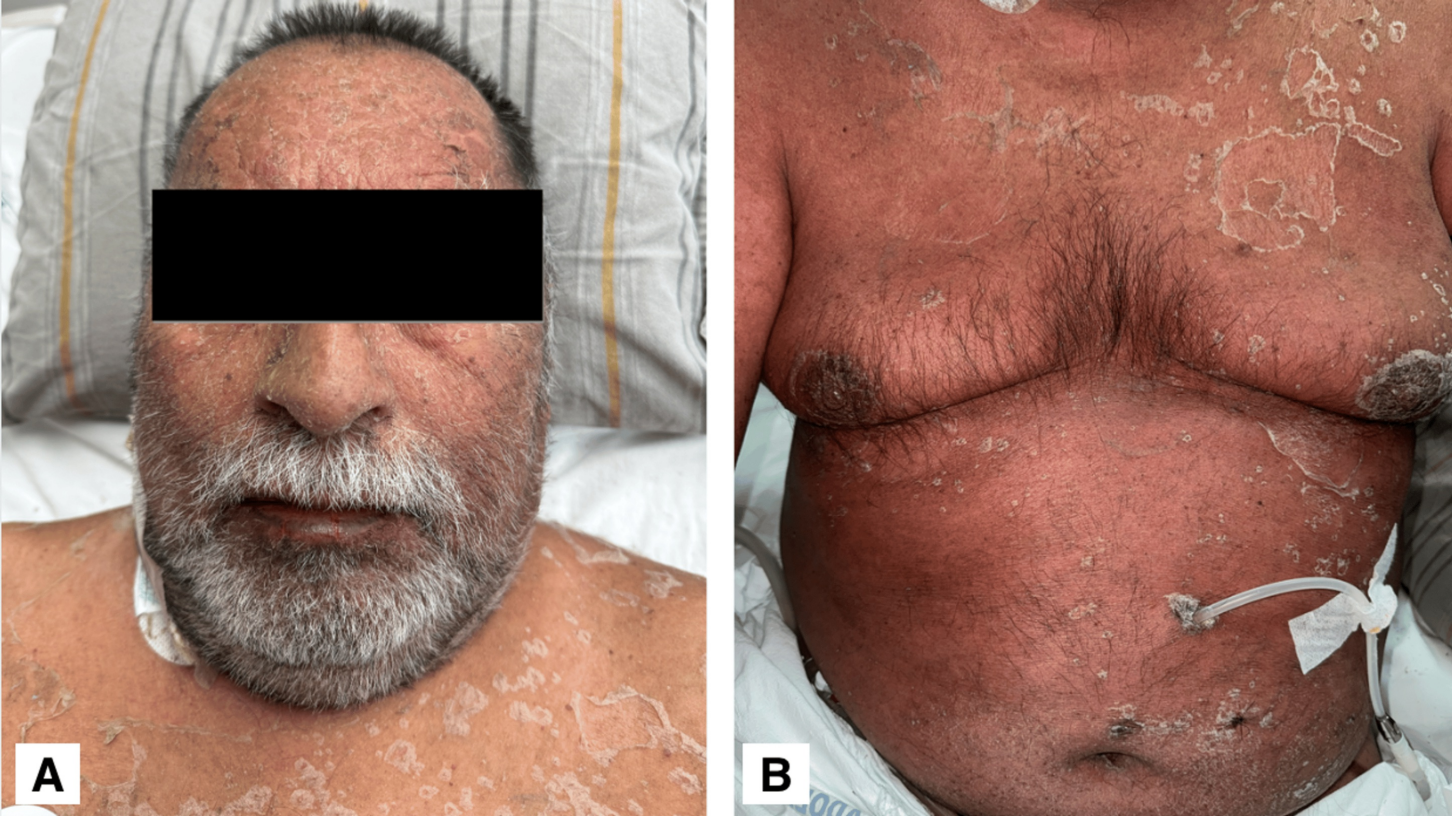
Scaly plaques diffusely distributed on the face, neck, anterior thorax, and abdomen. A. Diffuse desquamation involving the forehead, cheeks, eyelids, nose, lips, and beard area over an erythematous base. B. Superficial desquamation on the anterior chest and abdomen over an erythematous background.

Treatment was initiated with four doses of methylprednisolone 250 mg intravenously every 24 hours for three days, followed by prednisone at 1 mg/kg for three weeks. The patient responded favorably to the anti-inflammatory regimen, with improvement in cutaneous symptoms, resolution of pruritus, and reduction of erythematoviolaceous lesions, followed by generalized fine desquamation involving the face, trunk, and both upper and lower extremities (Figure 4). Laboratory parameters remained within target ranges, with noted improvement in liver function tests. In light of the clinical improvement, the patient was discharged with instructions for a gradual tapering of corticosteroids to minimize the risk of clinical relapse.

## Discussion

DRESS syndrome is a severe cutaneous adverse reaction triggered by certain drug families. Although rare, it is potentially fatal and characterized by a constellation of symptoms with variable onset and progression, including generalized rash, fever, hematologic abnormalities (leukocytosis, eosinophilia, and atypical lymphocytes), lymphadenopathy, and multiorgan involvement. It was first described in the 1930s, alongside the introduction of aromatic antiepileptics, the initial drug class associated with this reaction [1]. Currently, several drug families are recognized as potential triggers. Antiepileptics are the most commonly implicated group (37.3%), including both aromatic and non-aromatic agents, such as carbamazepine, lamotrigine, phenobarbital, phenytoin, and valproic acid; antibiotics are the second most frequent group (24.8%), particularly minocycline, vancomycin, beta-lactams, sulfonamides, and dapsone. Other associated agents include xanthine oxidase inhibitors (e.g., allopurinol, 8.2%), some antivirals (5.6%), particularly nevirapine, and certain antineoplastic and nonsteroidal anti-inflammatory drugs [1,2].

Clinically, DRESS typically presents with the abrupt onset of fever and intense pruritus, followed by a coalescing, confluent erythematous maculopapular eruption, usually starting on the trunk and extending to the face, neck, and upper and lower extremities, generally sparing palms and soles. The rash is often polymorphous, presenting with urticarial, purpuric, erythrodermic, eczematous, dyshidrosiform, exfoliative, vesiculobullous, pustular, lichenoid, or targetoid features [1]. Facial erythema and edema are reported in approximately 76% of cases and are considered sensitive and reliable indicators for suspecting DRESS. Mucosal involvement is typically mild and primarily affects the oral cavity and lips [1,2]. Cervical, axillary, and inguinal lymphadenopathy are frequently observed, as well as salivary gland edema; in some cases, xerostomia has been reported, which can hinder food and fluid intake [3]. Symptoms generally occur 2-12 weeks after drug exposure and are independent of the administered dose; they may arise with initial or subsequent exposures [4]. Following discontinuation of the culprit drug, a paradoxical worsening of symptoms may occur within the first three to four days, potentially mimicking an infectious process [3]. Hence, cautious use of antibiotics and antipyretics is advised, reserving them for confirmed infections to minimize cross-reactivity.

Systemically, DRESS frequently involves hematologic, hepatic, renal, pulmonary, and cardiac systems. Hematologic involvement predominates, characterized by fever, leukocytosis, eosinophilia, atypical lymphocytes, and lymphadenopathy. Hepatic involvement occurs in approximately 70-90% of cases, often presenting with elevated transaminases and a risk of progression to hepatic necrosis and acute liver failure [1,2]. Renal impairment is also significant, typically presenting with elevated creatinine and blood urea nitrogen, with a high risk of progression to chronic kidney disease [1,2].

In the case presented, the clinical picture was pronounced, allowing for early suspicion of DRESS. It was characterized by a generalized erythematous-violaceous, confluent maculopapular rash with poorly defined borders, symmetrically distributed, and associated with recent drug exposure. The patient had a history of stage V CKD (KDIGO) on peritoneal dialysis and had a peritoneal catheter. A recent peritoneal infection ("bacterial peritonitis") was documented, treated with intraperitoneal vancomycin 25 days prior to hospital admission, with no prior history of vancomycin exposure. Investigation revealed prior exposure to other high-risk medications, including nonsteroidal anti-inflammatory drugs (NSAIDs), atorvastatin, and vancomycin. Epidemiologically, antibiotics are the second most common drug class associated with DRESS, with glycopeptides, such as vancomycin, being implicated in this case [1]. Of note, the drug was administered via an atypical route: intraperitoneal. Meta-analyses and pharmacovigilance studies estimate the incidence of DRESS between 1/1,000 and 1/10,000, depending on the drug and geographic region; for vancomycin, the estimated incidence is 0.27/1,000 [5]. The average latency period for symptom onset is approximately 21 days, with a range of 10-32 days in cases with peripheral eosinophilia [5,6].

There are limited data and case reports on DRESS associated with vancomycin, which hampers understanding of its pathophysiology and classification [5]. It was previously linked to the HLA-A*32:01 allele; however, this association is not consistently observed across cases and may be influenced by ethnicity, particularly in Caucasian populations [5]. DRESS is more frequent in patients with systemic accumulation of reactive metabolites, especially those with pre-existing renal or hepatic dysfunction [4]. The cutaneous eruption is generally similar regardless of the drug; however, renal involvement is reported in approximately 75% of vancomycin-induced cases [6], noting that vancomycin is inherently nephrotoxic. Some case reports document significant improvement following discontinuation of the drug and initiation of systemic corticosteroids, suggesting immune-mediated interstitial nephritis as a possible mechanism [6]. Hepatic involvement is frequent, affecting 75-94% of patients [4], and may be accompanied by delayed transaminase elevation, which can lead to underdiagnosis; therefore, regular hepatic monitoring is recommended [7]. In the acute phase, liver enzymes may be elevated in 70-80% of cases, associated with hepatocellular damage, cholestatic patterns, and, less frequently, acute liver failure [4].

DRESS occurs more frequently in patients with systemic accumulation of reactive metabolites, particularly in those with pre-existing renal or hepatic disease [4]. Patients with reduced glomerular filtration rates tend to have higher vancomycin levels, which may increase the risk of DRESS [7]. The latency period for vancomycin-induced DRESS generally ranges from two to four weeks after drug initiation, regardless of the route of administration. Intraperitoneal administration does not initially produce high plasma vancomycin levels due to the slow absorption rate across the peritoneal membrane, which depends on membrane permeability. Its bioavailability after a four to six-hour dwell is approximately 30-70% compared to intravenous administration, which achieves 100% bioavailability, limiting a rapid rise in plasma concentrations. However, intraperitoneal administration results in sustained plasma concentrations, explaining its potential for systemic toxicity, particularly in patients with chronic kidney disease. To date, there is no clear association between plasma vancomycin levels and the incidence of DRESS. For future cases, strict monitoring of vancomycin plasma levels is recommended to better establish a causal relationship [6,7,10].

Regarding clinical manifestations, diagnostic scoring was performed using the RegiSCAR criteria, the most commonly used tool to confirm or rule out DRESS, with a score of 7 indicating a definitive case [2-4]. Additional diagnostic tests were pursued, including a peripheral blood smear that showed eosinophilia and atypical lymphocytes, liver chemistry abnormalities (with transaminase levels exceeding twice the upper limit of normal), and hepatomegaly on ultrasound. Based on these findings, treatment with corticosteroids was initiated, resulting in significant clinical improvement. Identification and discontinuation of the culprit drug is the cornerstone of management. First-line treatment consists of systemic corticosteroids, either administered intravenously or orally, at an initial dose of 1-2 mg/kg/day of prednisone or an equivalent, followed by gradual tapering due to the high risk of relapse after abrupt discontinuation [8]. The use of pulse-dose methylprednisolone remains controversial and is typically reserved for severe cases involving two or more organ systems, due to reports of adverse events including CMV reactivation, persistent symptoms, and increased mortality compared to oral therapy. Steroid-sparing agents are reserved for use in refractory disease with a poor response to corticosteroids. Case reports describe shorter hospitalization, symptom resolution, and biochemical remission with IV or oral cyclosporine (3-5 mg/kg/day in two divided doses for seven days, followed by a taper to 1.5-2.5 mg/kg/day for an additional seven days) compared to corticosteroid therapy, with lower relapse rates. Further studies are needed to validate its use, as renal toxicity currently limits its use in patients with established renal disease. JAK-STAT inhibitor tofacitinib has shown promising results in case reports at daily doses of 5-10 mg, reducing steroid dependency, controlling systemic and cutaneous symptoms, significantly lowering eosinophil counts, and achieving faster remission [8]. Strengthening medical training on the disease and its diverse clinical manifestations remains essential for early diagnosis and timely treatment, ultimately reducing associated complications and mortality.

## Conclusions

DRESS syndrome is a rare, underdiagnosed, and potentially fatal adverse drug reaction that is usually associated with intravenous drugs such as vancomycin. It requires a high level of clinical suspicion for early diagnosis and timely treatment, thereby significantly reducing associated sequelae and mortality. Despite DRESS associated with intravenous vancomycin being seen, reported cases following intraperitoneal administration are rare. This route of administration, commonly used in patients on peritoneal dialysis, can generate stable plasma concentrations leading to systemic toxicity. The clinical case presented highlights the importance of recognizing intraperitoneal vancomycin as a trigger, especially in patients with renal failure, in whom drug clearance is compromised. Given the rarity of cases involving the use of intraperitoneal drugs, coupled with the nonspecific and late presentation of this syndrome, physicians should always maintain a high level of suspicion, especially in patients exposed to well-identified high-risk medications such as vancomycin. Increased awareness of DRESS syndrome among general practitioners, peritoneal dialysis providers, and dermatologists allows for timely treatment, potentially preventing disease progression, reducing complications, and improving patient outcomes.
